# Radiotherapy for Metastatic Merkel Cell Carcinoma: A Review of the Literature

**DOI:** 10.1155/2012/654981

**Published:** 2012-07-02

**Authors:** Luluel Khan, Elizabeth A. Barnes

**Affiliations:** ^1^Princess Margaret Hospital, 610 University Avenue, Toronto, ON, Canada M5G 2M9; ^2^Department of Radiation Oncology, Odette Cancer Centre, Sunnybrook Health Sciences Centre, 2075 Bayview Avenue, Toronto, ON, Canada M4N 3M5

## Abstract

*Introduction.* Merkel cell carcinoma is a rare form of non-melanoma skin cancer of neuroendocrine origin. Optimal management of patients is controversial and the role of radiotherapy is unclear. *Purpose.* The purpose of this study was to review the efficacy of RT in the treatment of both local and distant metastatic disease from MCC. *Methods.* A literature search was conducted in MEDLINE (1946—January Week 1 2012) and Embase (1980–2012 Week 2). Articles of interest analyze the efficacy of radiotherapy for treatment of metastatic MCC and did not exclude case reports. *Results.* All articles except one focusing on the role of radiotherapy were of retrospective origin or case series. Significant limitations applied in all studies due to limited sample sizes and the retrospective nature of these studies. Radiotherapy improves locoregional control in the adjuvant setting, and many series suggest an improvement in overall survival. In cases where surgery is not possible, definitive radiotherapy may be an as-efficacious alternative. The radiosensitive nature of MCC coupled with existing reports suggests that treatment via current protocols for other primary tumors is adequate. *Conclusion.* Further studies should be conducted prospectively to clarify the true role of radiotherapy in metastatic MCC.

## 1. Introduction

Merkel cell carcinoma (MCC) is a rare form of nonmelanoma skin cancer of neuroendocrine origin which usually is found in elderly patients, in sun exposed skin (head and neck), and an increased incidence is seen with immunosuperessed patients [1]. The incidence of MCC according to SEER has risen to 0.44 per 100 000 in 2001, from 0.15 per 100 000 in 1986 [2]. In 2008 the Merkel Carcinoma Polyomavirus was identified and its role in pathogenesis is being investigated [3]. 

Although approximately 70% of patients with MCC present with stage I or II disease [4–6], low 5-year survival rates (reported to be 30%–64%) [7, 8] are attributed to high rates of locoregional and distant recurrence [9]. Time to recurrence is consistently reported as occurring at a median of 8 months [4, 5, 9, 10]. Along with lymph nodes, common sites of metastases include intransit skin, lung, CNS, bone, and liver [11]. The testis, pancreas, heart, prostate, GI tract, and bladder have been reported as sites of MCC metastases in the literature [11].

Treatment of MCC is primarily surgery, with adjuvant radiotherapy (RT: to which the disease is sensitive to) becoming more common, as recent data have shown RT improves both locoregional control and survival [5, 12, 13]. In areas where resection is not possible or where surgery is refused, RT alone is offered, and though data with this respect is relatively limited high rates of local control have been reported [14–16]. Sentinel lymph node biopsy (SLNB) is becoming standard practice for patients with clinically negative nodes. For clinically node-positive disease, typically node dissection followed by radiotherapy is delivered although primary radiotherapy may again be an option. As a rare disease, randomized, prospective data of various treatments for MCC are unavailable and most data on optimal treatment in the literature are supported by retrospective studies or case series. Further, robust data on the use of palliative RT specifically for metastases from MCC is severely lacking. The purpose of this paper is to describe the various uses of RT for managing both local and distance metastases from MCC. 

## 2. Methods

A literature search was conducted in MEDLINE (1946—January Week 1 2012) and Embase (1980–2012 Week 2). Broad search terms were utilized, including “merkel cell carcinoma”, “radiotherapy”, [“metasta*” or “recurrence” or “palliation”]. Papers selected included those focusing on the role of radiotherapy as adjuvant treatment of local metastases and as treatment alone for local and distant metastases. Case reports were also included due to the relatively few published reports on this topic. 

## 3. Results

The majority of included papers involved retrospectively analyzed data or case series. A single phase II, prospective trial of patients with MCC was found and investigated the addition of chemotherapy to adjuvant RT in stage I and II MCC. Jouary et al. prospectively compared regional adjuvant RT to observation in patients with stage I MCC [17]. 

### 3.1. Radiation as Adjuvant Nodal Treatment

Optimal avenues of treatment of nodal disease have been broadly divided by patients who have clinically node-negative disease versus those who are clinically node-positive. For clinically node-negative patients the rate of regional nodal relapse is high (50–66%) if untreated [17], and therefore lymph node dissection or prophylactic radiotherapy to the regional nodal bed was often recommended. Sentinel lymph node biopsy is now becoming widely accepted into routine clinical practice and is recommended in the NCCN guidelines. For patients with negative SLNB, Mehrany et al., found 97% (39/40) had no recurrence with omission of RT [18], and Gupta reported an 80% 3-year relapse free survival rate which did not alter with or without the use of RT [19]. Routine RT may be therefore be omitted in this population; however, there is a suggestion in the head and neck region that false negative SLNB may be seen due to aberrant patterns of lymphatic drainage [20]. Patients with positive SLNB traditionally undergo completion dissection followed by radiotherapy, although radiotherapy alone could be argued as an option. 

Surgery is recommended for clinically node-positive disease. Compared to node dissection alone, Veness found regional control was improved 2-fold with the addition of RT (37% versus 18%) [21]. In another retrospective review by Allen et al., risk of recurrence was 14% with surgery and 13% with surgery plus RT [4]. Fang et al. reported similar rates of regional recurrence in clinically node positive patients treated with CLND ± RT of 14% [22]. In nonresectable nodal disease, RT doses up to 60 Gy are recommended [23]. Optimal dose for definitive RT has yet to be defined and there is a paucity of data on dose response rates. For palliation, NCCN guidelines suggest a dose of 30 Gy in 10 fractions, though the data on which this recommendation is based was not given [24]. 

### 3.2. Radiation Alone for Nodal Treatment

Only a single study to our knowledge has investigated the potential role of RT alone for treatment of nodal involvement from MCC. Fang et al. prospectively collected data from patients with MCC over a 22-year period in the United States, which included patients who received RT alone for positive nodes [22]. Stratifying by microscopically involved lymph nodes (*n* = 26) versus clinically positive lymph nodes (*n* = 24), the authors assessed the role of radiotherapy alone versus complete lymphadenectomy (CLND) ±RT. In the group with microscopic disease, 100% regional control was obtained regardless of treatment (*n*_CLND±RT_ = 7, *n*_RT  alone_ = 19). In those with clinically positive lymph nodes, no significant difference was observed in 2-year recurrence free survival ± RT (*P* = 0.8). Those who received RT alone achieved a 2-year recurrence free survival of 78%  (*n* = 9) versus 73% in those who received CLND ± RT (*n* = 15). The authors concluded that RT provided similar rates of local control for node metastases from MCC and may be an option for patients given clinical factors and patients' desires. Their study also affirms that early detection of MCC metastases improves local control. Although criticisms regarding the retrospective nature of the study as well as relatively low sample size have been expressed in an editorial by Bichakjian et al. [25], Fang et al. provide the best available data in the literature regarding radiotherapy alone for nodal metastases from MCC in the absence of randomized trials.

Boyle in their retrospective series reported on 16 sites in 12 patients who received RT without surgery (3 of whom also received chemotherapy) for clinically determined nodal involvement [26]. Subsequent recurrence was not reported; however, 5 sites obtained a complete response and 7 obtained a partial response. Pacella et al. describe a series of patients receiving RT for MCC [27]. Eight patients received RT alone for regional lymph node involvement with 5 that attained a complete response, 1 patient with a partial response and the other two patients with complete response. 

Overall, data regarding use of RT alone for nodal involvement is sparse and limited by sample size and the retrospective nature of such studies. Similarly, much of the data was collected decades ago, demonstrating the need for current and ongoing research in this area. The question of what is the optimal dose for definitive RT was not examined in the Fang paper nor was a recommendation for dose/fractionation given. Given this is very radio responsive disease, dose escalation may not be required for bulky disease, and by defining what dose gives acceptable rates of local control in the adjuvant and definitive setting, the acute and long term toxicity of RT could be minimized.

### 3.3. Radiotherapy for Bone Metastases from MCC

Bone metastases represent approximately 10% of distant malignancy from MCC and are more commonly observed in the skull and less commonly so to the appendicular skeleton [28]. Palliation of bone metastases from MCC is efficacious due to both the radiosensitivity of the disease, plus the general efficacy of RT for bone metastases. Similar to treatment of bone metastases from other solid tumour primary cancers, treatment schedules can vary greatly in both dose and fractions given. In a case reported by Kamijo et al., a patient presented right hip pain subsequently determined to be metastatic MCC was treated with 30 Gy in 15 fractions postoperatively [29]. Unfortunately, other case reports did not detail RT doses.

Five cases of metastatic MCC to the spine have been reported to our knowledge in the literature [30–34]. In many cases, malignancy was accompanied by neurologic deficits caused by spinal cord compression and urgent surgery was required. Radiotherapy was efficacious in a number of cases; however, all patients succumbed to rapid disease progression.

### 3.4. Radiotherapy for Brain Metastases from MCC

Feletti et al. report a case of pituitary metastasis from MCC [35]. The patient was treated with stereotactic radiosurgery with a 25 Gy total dose in 3 fractions, combined with cisplatin and VP16. The patient was alive after 8 months, but visual impairments remained. Feletti et al. also provide a review of 14 previous cases in the literature of patients with brain metastases from MCC [35]. Of the 7 that received RT, 6 received whole brain radiotherapy while one received whole brain plus radiosurgery. Surgery and chemotherapy were prescribed to two patients each for the brain metastases. The radiotherapy doses and techniques utilized in these cases are similar to the treatment courses of patients with brain metastases from other solid tumors. Similarly, surgical and chemotherapeutic methods are dependent on patient and tumor characteristics. 

### 3.5. Radiotherapy for Other Metastases from MCC

Though metastases to other organs, such as, the prostate, bladder, liver, and kidneys, have been described in the literature [36] therapeutic interventions, particularly pertaining to radiotherapy specifics are rarely documented. As radiosurgery becomes more popular for treatment of metastatic disease, this certainly becomes an avenue of interest especially for such patients with relatively few visceral areas of disease, especially if surgery is not indicated.

In the case of cutaneous metastases from Merkel cell, where external beam radiotherapy may not be feasible due to size or location of the target, brachytherapy using a surface applicator maybe considered. In a case report by Cotter et al., surface-mold computer-optimized high-dose-rate brachytherapy was utilized to treat multiple cutaneous metastases in the lower extremity of a patient with a history of peripheral vascular disease. A rapid and durable treatment response was seen, a single recurrence within the treated area occurred at 25 months in the setting of diffuse metastatic disease [37].

## 4. Discussion

Treatment of primary Merkel cell carcinoma is well defined, with the mainstay being surgery accompanied by adjuvant radiotherapy [38]. Sentinel node dissection being increased used to assess regional nodal involvement [24]. As the disease progresses, guidelines become more controversial, especially pertaining to the role of radiotherapy as a primary treatment. Such data in the literature is confounded by both the retrospective nature of the studies in addition to small sample sizes, characteristic of rare diseases. Based on available studies, RT plays a key role in improving local control in nodal disease and is efficacious in palliating metastases to the bone, brain, and other organs. RT alone for management of local nodal metastases is suggested to provide similar rates of control to surgery, if the patient is not amenable to excision. 

The poor life expectancy observed in patients with MCC is a result of the aggressive nature of the disease and the high rate of metastases. Though no randomized trials have been conducted, observational data supports postoperative adjuvant radiotherapy [39]. Clark et al. showed that although adjuvant radiotherapy did not confer an improvement in disease-free survival in all stages, subset analysis showed that stage II patients demonstrated both improved DFS and DSS with adjuvant radiotherapy. When divided into stage IIa and IIb, patients without nodal metastases derived the greatest benefit. There was also a nonsignificant difference in DFS for stage I disease with adjuvant radiotherapy [40]. The importance of this stage-dependent finding is that patients who may be considered to have relatively low-risk disease (stage II) and hence may not be recommended for adjuvant radiotherapy, in fact appear to be the group that derive the greatest benefit. Although (stage III) did not show a benefit this was more likely due to low numbers of these patients. Thus the authors recommended adjuvant radiotherapy in both stage II and III patients. In addition, a recent meta-analysis demonstrated that in patients who received surgery and were deemed to have clear margins, adjuvant radiotherapy significantly improved local and regional recurrence (12% versus 39% and 23% versus 56%, resp.) [13]. Though a trend in survival increase was observed, this did not reach statistical significance. 

 The available literature tends to support adjuvant radiotherapy in management of nodal metastases [4, 21] though NCCN guidelines suggest that sentinel node dissection ± radiotherapy is another option [24]. Though data is unclear at this point, radiotherapy alone may provide similar rates of local control compared to surgery when the latter is not an option [22]. It should be noted that in primary MCC tumors treated with RT only, Mortier et al. found no difference in overall and disease-free survival compared to patients treated with surgery and adjuvant RT [15]. 

Perhaps more importantly beyond these results is the importance of early treatment and detection of MCC. Stage of disease at presentation is highly prognostic, with lower tumor burden associated with better outcomes. The subgroup of patients with small primary disease and SLNB-negative disease have good outcomes (97% with no recurrence at 7.3 months median followup) [18]. 

An important consideration in the many uses of radiotherapy for management of MCC is the balance of expected benefit and side effects. If for example, adjuvant treatment with radiotherapy only slightly improved local control in certain cases, are the associated morbidities and side effects worth the anticipated benefits? As MCC most commonly invades the upper regions of the body, considerations should be made regarding potential dysphagia, dental problems, xerostomia, loss of appetite, and weight loss [25]. Similarly, adjuvant irradiation of nodal metastases may result in lymphedema and cause further problems. More robust data regarding the role of radiotherapy is necessary to determine optimal strategies.

Palliation of bone, brain, and other visceral metastases from MCC is anticipated to be beneficial due to evidence from other solid tumors and the radiosensitivity of the disease; reports in the literature are based only on case reports. In patients with bone metastases, surgery may be warranted in some patients, as observed in the reports where metastatic tumors result in neurological deficits, and a wealth of guidelines exist on this topic which are likely applicable to patients with MCC especially in the absence of such data for this group. Brain metastases may similarly be resected or treated conventionally with palliative radiotherapy or more aggressively with stereotactic radiosurgery. Again, guidelines exist in the literature-depicting scenarios where one may prove more advantageous over the other.

Combination of chemotherapy with radiotherapy and surgery has also been evaluated with conflicting findings. TROG 96:07 is the only phase II, prospective trial to date that has evaluated outcomes with specific treatments for patients with MCC [41]. The authors concluded that combination carboplatin, etoposide, and RT did not improve survival in patients compared to historical control. An earlier study suggested that chemotherapy for recurrent or advanced disease may be of benefit to patients with good performance status [42]. Whether or not systemic treatments are beneficial remains to be seen, though a number of trials have been registered with a variety of interventions at time of writing.

The data presented are limited by issues common to research in rare diseases. As observed in all studies, data were retrospectively collected and therefore, other details, such as, concomitant systemic treatments were in most cases unavailable and may have confounded these results. Similarly, due to small sample sizes, a lack of robust data results in the inability to draw strong conclusions regarding optimal management. Further, smaller health centers may observe few, if any, cases of MCC, and as such, the presented data may be biased towards tertiary reports. 

To conclude, radiotherapy plays an important role in the management of both local and distant metastases from MCC. It potentially improves local control as adjuvant treatment of nodal disease, or by itself when excision is not possible. Palliation of bone, brain and other systemic metastases can be primarily via radiotherapy and is assumed to be efficacious due to the radiosensitive nature of the disease and the fact that such treatment is standard when metastases result from other primary cancers. Further prospective data should be collected to better characterize the role of radiotherapy under varying circumstances.
